# Effects of kinetics of light‐induced stomatal responses on photosynthesis and water‐use efficiency

**DOI:** 10.1111/nph.14000

**Published:** 2016-05-23

**Authors:** Lorna McAusland, Silvère Vialet‐Chabrand, Philip Davey, Neil R. Baker, Oliver Brendel, Tracy Lawson

**Affiliations:** ^1^ School of Biological SciencesUniversity of EssexColchesterCO4 3SQUK; ^2^ INRAUMR1137 ‘Ecologie et Ecophysiologie Forestières’F‐54280ChampenouxFrance; ^3^ UMR1137 ‘Ecologie et Ecophysiologie Forestières’Faculté des SciencesUniversité de LorraineF‐54500Vandoeuvre‐Les‐NancyFrance

**Keywords:** guard cells, intrinsic water use efficiency, kinetics of stomatal responses, photosynthesis, stomatal conductance

## Abstract

- Both photosynthesis (*A*) and stomatal conductance (*g*_s_) respond to changing irradiance, yet stomatal responses are an order of magnitude slower than photosynthesis, resulting in noncoordination between *A* and *g*_s_ in dynamic light environments.
- Infrared gas exchange analysis was used to examine the temporal responses and coordination of *A* and *g*_s_ to a step increase and decrease in light in a range of different species, and the impact on intrinsic water use efficiency was evaluated.
- The temporal responses revealed a large range of strategies to save water or maximize photosynthesis in the different species used in this study but also displayed an uncoupling of *A* and *g*_s_ in most of the species. The shape of the guard cells influenced the rapidity of response and the overall *g*_s_ values achieved, with different impacts on *A* and *W*_i_. The rapidity of *g*_s_ in dumbbell‐shaped guard cells could be attributed to size, whilst in elliptical‐shaped guard cells features other than anatomy were more important for kinetics.
- Our findings suggest significant variation in the rapidity of stomatal responses amongst species, providing a novel target for improving photosynthesis and water use.

## Introduction

Stomata control the balance of gases between the internal leaf environment and the external atmosphere; regulating CO_2_ uptake for photosynthesis and water loss through transpiration (*E*). Low stomatal conductance to water vapour (*g*_s_) can restrict CO_2_ uptake by limiting CO_2_ influx and thus net CO_2_ assimilation rate (*A*), whereas high *g*_s_ facilitates high rates of *A,* but greater water loss is an inevitable consequence.

This balance between CO_2_ limitation and water loss is characterized by intrinsic water use efficiency (*W*_i_), which is the ratio between *A* and *g*_s_. On an instantaneous timescale, maintaining a suitable and appropriate balance is impeded by the temporal stomatal responses, which are a magnitude slower than those of *A*. Therefore, in response to changing light, the kinetics of *g*_s_ can greatly impact CO_2_ uptake and water loss, which has significant implications water use efficiency (WUE). WUE can be defined as the ratio of net CO_2_ uptake relative to water loss through transpiration (E) or as the ratio of biomass or yield accumulation to water use over the growing season. Consequently, WUE is often a target for improving crop performance; however, it should be noted that greater *W*_i_ is often at the expense of *A* (Blum, 2009; Lawson *et al*., 2010; Lawson & Blatt, 2014). The rate of water transpired through the stomata is an order of magnitude greater than the rate of CO_2_ uptake for *A* due to the greater water concentration gradient between the intercellular spaces within the leaf and the external atmosphere (as well as biochemical limitation on *A*). In order to maintain an optimal balance between *A* and *E*, stomatal guard cells are continually adjusting to environmental and intracellular cues (Lawson & Blatt, 2014).

Many previous studies have reported a strong correlation between *A* and *g*_s_ (Wong *et al*., 1979; Farquhar & Sharkey, 1982). This correlation is generally observed because steady‐state values are often reported, yet under dynamic conditions *g*_s_ responses are not always coupled with *A* (Knapp & Smith, 1987, 1990). In natural environments, photosynthetic photon flux density (PPFD) fluctuates on timescales of seconds to days and seasons (Assmann & Wang, 2001) driven by changes in cloud cover, sun angle and shading from adjacent leaves in the canopy (Pearcy, 1990; Chazdon & Pearcy, 1991; Way & Pearcy, 2012). Plants therefore experience short and long term fluctuations in PPFD creating ‘sun’ and ‘shade’ flecks to which *A* and *g*_s_ respond. Slower stomatal opening when *A* responds rapidly to a PPFD increase can limit CO_2_ assimilation (Tinoco‐Ojanguren & Pearcy, 1993), whilst delayed stomatal closing responses following a decrease in PPFD and photosynthesis result in unnecessary water loss when carbon gain is limited (Lawson *et al*., 2010; Lawson & Blatt, 2014). Due to the difference in the rate of carbon gain to water loss, any disparity in the response of *A* and *g*_s_ also increases the probability of water stress (Condon *et al*., 2002). For example, slow closing of stomata when *A* has decreased will result in higher than necessary transpiration rates that will deplete the soil water more rapidly and thus potentially create a soil water deficit.

It has been estimated that stomata can limit *A* by up to 20%, which can impact substantially on crop yields (Farquhar & Sharkey, 1982; Jones, 1987, 1998; Fischer *et al*., 1998; Lawson & Blatt, 2014). In order to maximize *A* and optimize *W*_i_, species or cultivars with rapid stomatal responses would be intuitively desirable, as there would be greater synchrony with mesophyll demands for CO_2_. Amplitude and rapidity of stomatal movements are therefore potential targets to improve *A* and *W*_i_. The majority of studies reporting stomatal influences on photosynthesis describe steady‐state values and explore the potential of increasing or decreasing *g*_s_ to enhance *A* or diminish water loss, however this often results in an overall reduction of *A* and thus productivity (Blum,^2009^). We propose here to follow another approach for plant improvement, which exploits stomatal kinetics to facilitate synchronous *g*_s_ responses with mesophyll demands for CO_2_, thus simultaneously reducing CO_2_ limitations as well as avoidable water losses, incidentally enhancing *W*_i_.

Only a handful of investigations have focused on dynamic stomatal responses and even fewer of these have explored the effects on *A* and *W*_i_. Most of these studies have examined forest understorey species and the impact of sun‐fleck regimes on *A* and *E* (Pearcy, 1990; Tinoco‐Ojanguren & Pearcy, 1993; Leakey *et al*., 2005; Way & Pearcy, 2012). In addition, assessing the rapidity of stomatal movements is complicated by variation in both the sensitivity and responsiveness of stomata between different species (Ooba & Takahashi, 2003; Lawson *et al*., 2010; Vico *et al*., 2011) and between individuals of the same species grown in different habitats (Drake *et al*., 2013). After a change in PPFD, the temporal response of *g*_s_ is usually composed of three steps: an initial lag where the value of *g*_s_ remains stable for several minutes, followed by an exponential phase during which rapid increases in *g*_s_ are observed before reaching final steady‐state plateau (Naumburg *et al*., 2001; Vialet‐Chabrand *et al*., 2013). Recently, a dynamic sigmoidal model has been developed by Vialet‐Chabrand *et al*. (2013) to analyse the temporal response of *g*_s_ by estimating the initial lag time (λ), a time constant (*k*) and a steady‐state target (*G*_smax_; see Table 1 for a summary of parameters and units). The time constant was used to describe the rapidity of the exponential phase independently of the amplitude of the *g*_s_ response, facilitating species or cultivar comparisons as well as proposing a more accurate interpretation.

**Table 1:** A summary of parameters referred to within the text with accompanying units

Parameter	Definition	Units
*A*	Net CO_2_ assimilation rate	μmol m^−2^ s^−1^
*A*_95_	95% maximum *A* under 1000 μmol m^−2^ s^−1^ PPFD	μmol m^−2^ s^−1^
*C*_a_	Atmospheric CO_2_ concentration	μmol mol^−1^
*C*_i_	Intracellular CO_2_ concentration	μmol mol^−1^
*E*	Water loss via transpiration	mol m^−2^ s^−1^
GCW	Guard cell width	μm
*g*_s_	Stomatal conductance to water vapour	mmol m^−2^ s^−1^
*G*_smax_	Predicted steady‐state *g*_s_ under 1000 μmol m^−2^ s^−1^ PPFD	mmol m^−2^ s^−1^
*G*_smin_	Predicted steady‐state *g*_s_ under 100 μmol m^−2^ s^−1^ PPFD	mmol m^−2^ s^−1^
*k*	Time constant describing time taken to achieve steady‐state g_s_	min
*k*_i_	Time constant for *g*_s_ to increase to *G*_smax_ under 1000 μmol m^−2^ s^−1^ PPFD	min
*k*_d_	Time constant for *g*_s_ to decrease to steady‐state under 100 μmol m^−2^ s^−1^ PPFD	min
PL	Stomatal pore length	μm
PPFD	Photosynthetically active photon flux density	μmol m^−2^ s^−1^
SD	Stomatal density	mm^−2^
*Sl*_max_	Maximum rate of *g*_s_ opening to an increase in PPFD from 100 to 1000 μmol m^−2^ s^−1^	mmol m^−2^ s^−1^
*r*_0_	Minimum *g*_s_ of the sigmoidal response of *g*_s_ to a step increase in PPFD	mmol m^−2^ s^−1^
VPD	Vapour pressure difference from leaf to air	kPa
*W*_i_	Intrinsic water‐use efficiency	μmol mol^−1^
*W*_i95_	*W*_i_ at *A*_95_	μmol mol^−1^
*W*_imax_	Maximum *W*_i_ under 1000 μmol m^−2^ s^−1^ PPFD	μmol mol^−1^
λ	Initial lag in the response time of *g*_s_ to a step increase in PPFD	min

In order to determine the impact of stomatal responses to increasing and decreasing PPFD on limitation of *A*, water loss and intrinsic WUE, we have assessed and quantified the rapidity of stomatal movements in a range of plant types, including several major crops. We have selected species with kidney‐ and dumbbell‐shaped guard cells to estimate the influence of anatomical features on rapidity of responses.

## Materials and Methods

### Plant material and growth conditions

Thirteen important crop species (including three C_4_ species) were selected along with the model plant *Arabidopsis thaliana,* and the relict gymnosperm species *Ginkgo biloba*. Eight had kidney‐ or elliptical‐shaped guard cells whilst four had dumbbell‐shaped guard‐cells that are typically found in grasses.

*Arabidopsis thaliana* (Columbia, Col‐0) seed was germinated in 100‐cm^3^ pots containing peat‐based compost (Levingtons F2S, Everris, Ipswich, UK) and grown in a controlled environment (Reftech BV, Sassenheim, the Netherlands). Photosynthetic photon flux density (PPFD) was maintained at 155 ± 10 μmol m^−1^ s^−1^ for an 8 h photoperiod, whilst temperature and vapour pressure deficit (VPD) were 23°C and 1.1 kPa, respectively, day and night.

Oat (*Avena sativa*), sunflower (*Helianthus annuus*), tobacco (*Nicotiana tabacum*), pea (*Pisum sativum)*, tomato (*Solanum lycopersicum*), Sorgum (*Sorghum bicolor*), Barly (*Hordeum vulgare*), wheat (*Triticum aestivum*), maize (*Zea mays*), French bean (*Phaseolus vulgaris*) and broad bean (*Vicia faba*) were germinated in 650‐cm^3^ pots containing peat‐based compost (Levington F2S). Following germination, plants were grown in a temperature‐controlled glasshouse for 4–8 wk before measuring. Established Miscanthus (*Miscanthus nepalensis*) were supplied in 1‐l pots from a commercial nursery (Beth Chatto, Colchester, UK). Solar radiation provided a PPFD of *c*. 500 μmol m^−2^ s^−1^, supplemented by sodium vapour lamps (600W; Hortilux Schrèder, Monster, the Netherlands) to 300 μmol m^−2^ s^−1^ PPFD when external PPFD dropped below 1200 μmol m^−2^ s^−1^ over a 10 h period. Air temperature was maintained at 25°C ± 3°C during the day and 18°C ± 3°C at night. Plants were watered daily from below, with any excess water not absorbed by the pot within 2 h removed.

Rice (*Oryza sativa*) seeds were germinated and transferred to 650‐cm^3^ pots as described above and grown in a controlled environment with a photoperiod of 12 h : 12 h, light : dark at a PPFD of 500 ± 20 μmol m^−2^ s^−1^, a day temperature of 25°C and VPD of 0.8 ± 0.2 kPa. Plants were measured after 12 wk.

### Leaf gas‐exchange measurements

Photosynthetic carbon assimilation (*A*) and stomatal conductance to water (*g*_s_) were measured on the youngest fully expanded leaf using infrared gas analysis (Li‐Cor 6400, Lincoln, NB, USA, and CIRAS‐1, PP Systems, Amesbury, MA, USA). Light was provided by an integrated LED light source (Li‐Cor, PP Systems). Leaves were first equilibrated at a PPFD of 100 μmol m^−2^ s^−1^ until both *A* and *g*_s_ reached ‘steady state’, this being defined as a < 2% change in rate during a 10‐min period (*c*. 30–60 min). Once steady state was satisfied, PPFD was increased to 1000 μmol m^−2^ s^−1^ for 1 h before returning to 100 μmol m^−2^ s^−1^ for 30 min. The leaf cuvette was maintained at 400 μmol mol^−1^ CO_2_ concentration (*C*_a_), a leaf temperature of 20°C (±2°C) and a VPD of 1 ± 0.05 kPa. *A* and *g*_s_ were recorded every 1 min. Intrinsic water use efficiency (WUE) was calculated as *W*_i_ = *A*/*g*_s_. All measurements were completed before 14:00 h to avoid any unwanted diurnal or circadian effects on photosynthesis.

### Leaf anatomical measurements

Stomatal impressions of the ad‐ and abaxial leaf surfaces were taken of the same area, measured using gas exchange. A negative impression was made using a dental polymer (Xantoprene, Heraesus Kulzer Ltd, Hanau, Germany) following the methods of Weyers & Johansen (1985). Once the impression material had dried and was removed from the leaf, a positive impression was made from this by placing in nail varnish on a microscope slide. Stomatal density, guard cell length (L) and guard cell width were determined using ImageJ software (National Institute of Health, Bethesda, MD, USA) from twenty fields of view (size 1250 μm^2^) captured from each impression using a 5 MP eye‐piece camera (MicroCAM 5 MP, Bresser Optics, Rhede, Germany).

### Modelling *g*_s_, *A* and *W*_i_ responses to PPFD

**Figure 1:**
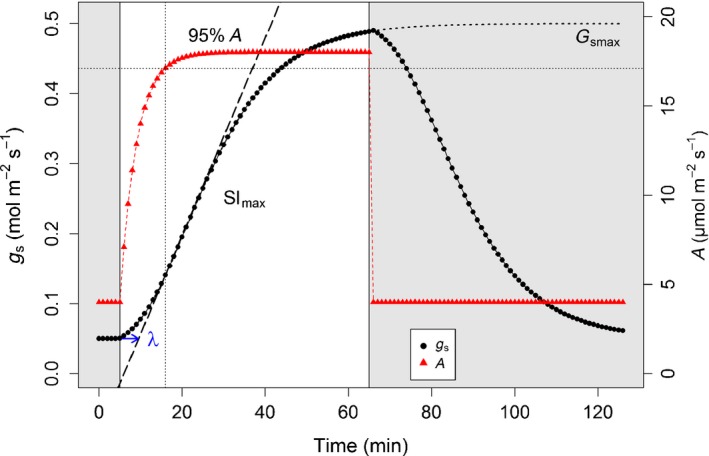
Theoretical temporal response of stomatal conductance (*g*_s_; black) and net CO_2_ assimilation (*A*; red) to a step change in PPFD from 100 (shaded area) to 1000 (unshaded area) μmol m^−2^ s^−1^. Where *Sl*_max_ describes the maximum temporal response of *g*_s_ (dashed line), λ describes the time‐lag before *g*_s_ starts to increase (blue arrow) and *G*_smax_ describes the steady‐state target of *g*_s_ under 1000 μmol m^−2^ s^−1^ PPFD. The dotted lines represented the time and the value were 95% *A* is reached.

In order to describe the temporal response of *g*_s_ to a single step‐change in PPFD, an analytical model derived from the model by (Vialet‐Chabrand *et al*., 2013) was used (Fig. 1).

The model described the temporal response of *g*_s_ using a time constant (*k*, min), an initial time lag (λ, min) and a steady‐state *g*_s_ (*G*_smax_, mmol m^−2^ s^−1^) reached at given PPFD:

(Eqn 1) $$g_{s}=\left(G_{s\;\max}-r_{0}\right)e^{-e^{\left(\frac{\lambda -t}{k}\;\;+\;\;1\right)}}+r_{0}$$

(*t*, time, where time 0 is the point at which PPFD was increased from 100 to 1000 μmol m^−2^ s^−1^; *r*_0_ (mmol m^−2^ s^−1^), initial value of stomatal conductance before the change in PPFD). In this equation, the time constant *k* is a measure of the rapidity of response of *g*_s_ independent of the amplitude of variation in *g*_s_ (Eqn 2). To distinguish between the time taken for the stomata to open (increase) and to close (decrease), the abbreviations *k*_i_ and *k*_d_ are used.

A second parameter combining rapidity and amplitude of the response, the maximum slope (*Sl*_max_), was used to describe the maximal slope of the *g*_s_ response to the step‐change in PPFD:

(Eqn 2) $$Sl_{\max}=\frac{k.\left(G-r_{0}\right)}{e}$$

Parameter values were estimated using a Metropolis Hasting algorithm and a Bayesian model. The priors (*a priori* probability of the parameter values) used were uniform covering a large range of possible values and the initial values were chosen randomly. The initial values were chosen from observed values (± 10%) of both *r*_0_ and *G*. For *k*, the range of values were selected from between 10 and 60 min, whilst λ values were between 0.1 and 5 min. After 100 000 iterations using a thinning factor of 15, the chains were checked for stability and convergence (see Table 1).

### Temporal responses in *g*_s_ limits *A*

During a step increase in PPFD, photosynthesis was considered limited by stomatal conductance until 95% *A* (*A*_95_) was reached. Using this assumption, the percentage of limitation of *A* by *g*_s_ was estimated by:

(Eqn 3) $$\text{Limitation}\;\left(\%\right)=\frac{\int_{0}^{t}\left(A_{\max}-A\right)}{\int_{0}^{60}A_{\mathrm{tot}}}$$

($\int_{0}^{t}\left(A_{\max}-A\right)$, integral of the difference between the maximum potential *A* (*A*_max_) and the observed limited *A* from the beginning of the observed curve to the time *t* where *A* reached 95% of the steady state; $\int_{0}^{60}A_{\mathrm{tot}}$, maximum integral of *A* for 1 h period). Calculating the ratio using $\int_{0}^{60}A_{\mathrm{tot}}$ normalized *g*_s_ limitation over the 1‐h measurement period (see Table 1 for a summary of parameters).

### The impact of different *g*_s_ and *A* responses on water loss

The nonsynchronous *g*_s_ and *A* response influences the temporal *W*_i_ response and the amount of water lost following a step increase in PPFD. To investigate the impact of *g*_s_ responses on water use efficiency, we predicted *g*_s_ from a simple model (*g*_s_ = *A*/*W*_i_) using a constant *W*_i_ during the transient response. The constant value of *W*_i_ was chosen close to the maximum *A* (95%), assuming that this would be close to an optimal *W*_i_ with no limitation of *A*. On the one hand, when observed values of *g*_s_ were greater than predicted by the constant *W*_i_ model, more water was ‘lost’ than required to maintain optimal *A;* on the other, when observed values of *g*_s_ were lower than predicted, water was ‘saved’, illustrating a close coupling of *g*_s_ with *A*. As an investigating tool, this approach allowed us to assess the percentage of water ‘lost’ and ‘saved’ by comparing the coupling between *A* and *g*_s_ in different species.

### Statistical analysis

Statistics were conducted using Spss (v.16; SPSS Inc., Chicago, IL, USA) and R (http://www.r-project.org/). A Shapiro–Wilk test was used to test for normality and a Levene's test of homogeneity was used to determine if samples had equal variance. Single factor differences were analysed using a one‐way ANOVA with a Tukey–Kramer honest significant difference test where more than one group existed or a Student's *t*‐test where only two groups were compared.

## Results

**Figure 2:**
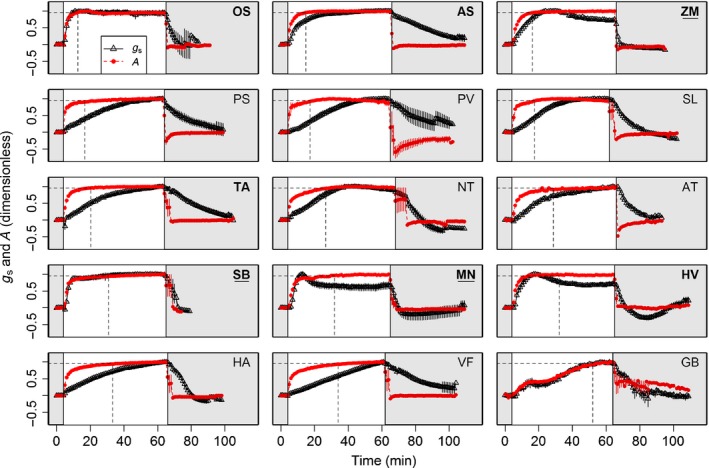
Normalized temporal response of net CO_2_ assimilation (*A;* circles) and stomatal conductance to water vapour (*g*_*s*_; triangles) of 15 species to an increase in irradiance from 100 (shaded area) to 1000 (unshaded area) μmol m^−2^ s^−1^ followed by a decrease to 100 μmol m^−2^ s^−1^ (see Table 3 for abbreviations of species nomenclature). The dashed line indicates where 95% maximum *A* (*A*_95_) was achieved. Data are the mean ± SE (*n* = 3–5). Values were normalized to the initial values at 100 μmol m^−2^ s^−1^ PPFD and maximum values at (1000 μmol m^−2^ s^−1^ PPFD).

**Figure 3:**
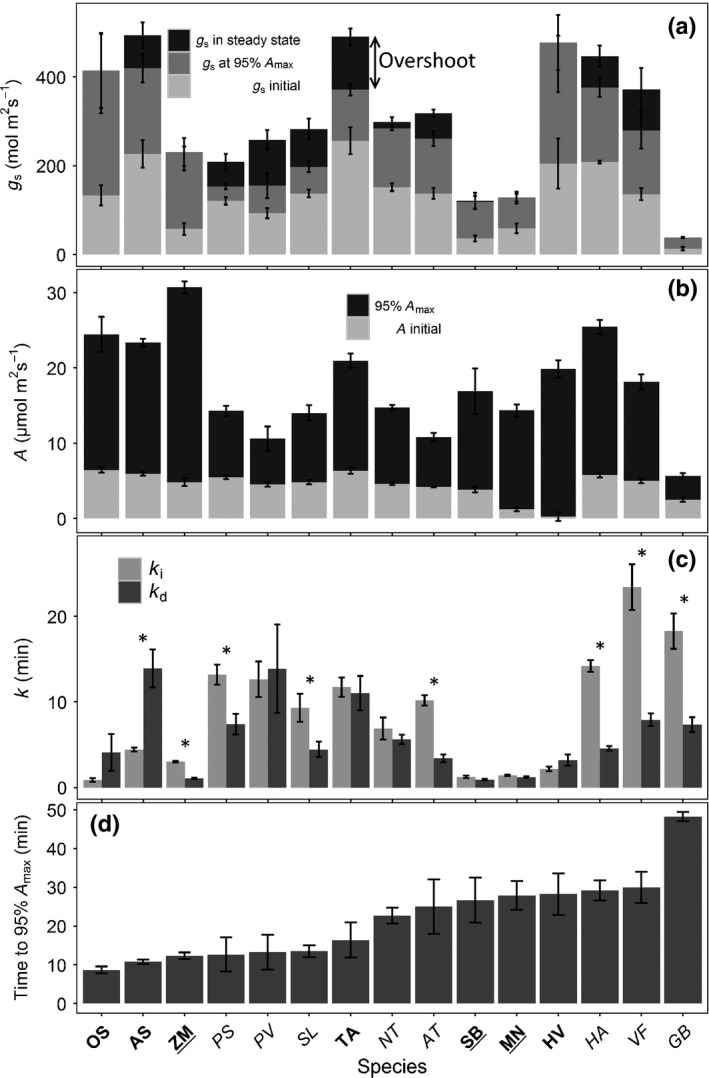
Comparison between species of (a) steady‐state *g*_s_ under 100 μmol m^−2^ s^−1^ PPFD (*G*_smin_), *g*_s_ at 95% maximum net assimilation under 1000 μmol m^−2^ s^−1^ PPFD (*A*_95_) and steady state (*G*_smax_) under 1000 μmol m^−2^ s^−1^ PPFD for 15 species; (b) steady‐state *A* under 100 μmol m^−2^ s^−1^ PPFD (*A* initial) and *A*_95_; (c) time constants k_i_ and k_d_ for stomatal opening and closure, respectively; and (d) the time taken to reach A_95_. Data are the mean ± SE (*n* = 3–5). Asterisks represented a significant asymmetry of *k*_i_/*k*_*d*_ (*P *<* *0.05). Species in bold have dumbbell‐shaped guard cells, underlined species have a C_4_ metabolism and species in plain font have elliptical‐shaped guard cells and C_3_ metabolism (see Table 3 for species name abbreviations).

Most species measured achieved steady‐state *g*_s_ after 60 min of high PPFD, except *Helianthus* and *Vicia*, which had not attained their maximum *g*_s_ values within this timeframe (Fig. 2), which might have led to an underestimation of their *k*_i_ values, which were already high (Fig. 3). Additionally, Ginkgo displayed atypical *g*_s_ and *A* behaviour (Fig. 2). The 30‐min exposure to low light may not have been sufficient for complete, steady‐state stomatal closure for some species; however, this does not greatly impact our estimations of additional water loss compared to instantaneous stomatal responses, because the major part of the water loss can be attributed to the initial rapid opening response of the stomata (Fig. 2).

### Quantifying *A* and *g*_s_ responses to step changes in PPFD

Steady‐state *g*_s_ at the initial PPFD of 100 μmol m^−2^ s^−1^ (*G*_smin_) varied significantly among the species (*F*_(14,49)_ = 5.007, *P *<* *0.0001), with the lowest values recorded for *G. biloba* (13.2 mmol m^−2^ s^−1^) and highest values for *T. aestivum* (255.9 mmol m^−2^ s^−1^) (Fig. 3a; Supporting Information Fig. S1), whereas *A* was below 9 μmol m^−2^ s^−1^ for all species (Figs 3b, S2). An increase in PPFD to 1000 μmol m^−2^ s^−1^ led to an immediate and rapid increase in *A* compared to *g*_s_ for all species. After this initial period, the increase in *A* slowed to a magnitude similar to the concurrent increase in *g*_s_. For the majority of species, *A* reached steady state while *g*_s_ continued to increase. These different periods of coupled and uncoupled responses of *A* and *g*_s_ were species‐dependent. Although the majority of species displayed a mainly uncoordinated *A* and *g*_s_ temporal response (Fig. 2), final steady‐state values of *A* and *G*_smax_ were significantly correlated among species (*r*_s(51)_ = 0.78, *P *<* *0.001 for C_3_ species and *r*_s(13)_ = 0.84, *P *<* *0.001 for C_4_ species – Fig. S3). In contrast to the majority of the species examined, *S. bicolor, O. sativa* and *G. biloba* all exhibited low *g*_s_ and an unusually strong coupling between *A* and *g*_s_. The key difference between these three species was that *S. bicolor* and *O. sativa* exhibited a faster response of *A* and *g*_s_, whereas *G. biloba* showed rather slower responses.

The steady‐state values of *g*_s_ (at 1000 μmol m^−2^ s^−1^ PPFD) estimated by the model (*G*_smax_) were significantly different among species (*P *<* *0.0001, *F*_(14,49)_ = 14.469; Fig. 3a), with observed values 10‐fold higher in *T. aestivum* (482.9 ± 18.7 mmol m^−2^ s^−1^) compared with *G. biloba* (45.2 ± 1.9 mmol m^−2^ s^−1^). Values of *G*_smax_ were positively related to *Sl*_max_ (*r* = 0.61, *P *<* *0.01) in elliptical‐ and dumbbell‐shaped guard cells (*r *=* *0.41, *P *<* *0.05), whereas *Sl*_max_ was related to the total time taken to open to *G*_smax_ (*k*_i_) in elliptical‐ (*r *=* *−0.54; *P *<* *0.01) and dumbbell‐shaped (*r *=* *−0.68; *P *<* *0.01) guard cells (Table 2). Six out of the seven species with dumbbell‐shaped guard cells showed the highest values of *Sl*_max_ (Table 3). Applying the Vialet–Chabrand model to the temporal response of *g*_s_ to an increase from 100 to 1000 μmol m^−2^ s^−1^ PPFD, showed that the initial lag time in the *g*_s_ response (λ) was significantly different among species (*P *<* *0.0001, *F*_(14,49)_ = 5.819) and ranged from 12 s for *A. sativa* to 6 min for *G. biloba* (Table 3).

**Table 2:** Correlation matrix between parameters (grey cells) describing the temporal response of *g*_s_ during opening and closing of elliptical‐ (upper triangle of the matrix) and dumbbell‐shaped (lower triangle of the matrix) guard cells (see Table 3)

	Elliptical						
Dumbbell	*Gs*_max_	ns	−0.48*	0.61**	0.47*	ns	ns
	ns	*k*_i_	ns	−0.54**	ns	ns	ns
	ns	ns	λ	ns	ns	ns	ns
	0.41*	−0.42*	ns	*Sl*_max_	ns	0.47*	0.46*
	ns	ns	ns	ns	SD	ns	ns
	0.47**	0.72***	ns	ns	ns	PL	0.91***
	ns	ns	ns	ns	0.55**	−0.41*	GCW

	Elliptical						
Dumbbell	*Gs*_max_	ns	ns	ns	ns	ns	ns
	0.44*	*K*_*d*_	ns	0.68***	ns	ns	ns
	ns	0.49**	λ	ns	ns	ns	ns
	0.37*	0.62***	ns	*Sl*_max_	−0.68***	ns	ns
	ns	ns	ns	ns	SD	ns	ns
	0.6***	0.73***	ns	ns	ns	PL	0.91***
	ns	ns	ns	ns	0.55**	−0.41**	GCW

*G*_smax_, predicted steady‐state *g*_s_ under 1000 μmol m^−2^ s^−1^ PPFD; *k*_i,_ time constant for *g*_s_ to **i**ncrease to *G*_*s*max_ under 1000 μmol m^−2^ s^−1^ PPFD; *k*_*d*_, decrease from *G*_smax_ to *G*_smin_ under 100 μmol m^−2^ s^−1^; λ, initial lag in the response time of *g*_s_ to a step increase in PPFD; *Sl*_max,_ maximum rate of *g*_s_ opening to an increase in PPFD from 100 to 1000 μmol m^−2^ s^−1^. Anatomical parameters of stomatal density (SD), pore length (PL) and guard cell width (GCW) were also compared. Significance: *, *P *<* *0.05; **, *P *<* *0.01; ***, *P *<* *0.001; ns, not significant.

**Table 3:** Parameters of the dynamic model of *g*_s_ as estimated from a step increase in irradiance from 100 to 1000 μmol m^−2^ s^−1^ for 15 species

Species	Shape of guard cell/metabolism	Graph initials	*K*_i (_min)	*K*_d (_min)	λ (min)	*Sl*_max_ (mmol m^−2^ s^−2^)	G (mmol m^−2^ s^−1^)
***Oryza sativa***	Dumbbell/C_3_	OS	0.9 ± 0.21^a^	4.1 ± 2.16^abc^	0.11 ± 0.02^a^	1.91 ± 0.60^a^	424.50 ± 89.99^abcd^
***Sorghum bicolor***	Dumbbell/C_4_	SB	1.2 ± 0.16^a^	0.9 ± 0.09^a^	1.04 ± 0.17^a^	0.46 ± 0.11^bc^	118.32 ± 15.91^ef^
***Miscanthus nepalensis***	Dumbbell/C_4_	MN	1.4 ± 0.11^a^	1.2 ± 0.10^a^	1.36 ± 0.16^ab^	1.56 ± 0.11^bc^	175.56 ± 18.53^ef^
***Hordeum vulgare***	Dumbbell/C_3_	HV	2.2 ± 0.30^a^	3.2 ± 0.70^ab^	0.62 ± 0.37^a^	1.01 ± 0.24^ab^	529.07 ± 55.85^a^
***Zea mays***	Dumbbell/C_4_	ZM	3.0 ± 0.10^ab^	1.1 ± 0.08^a^	1.37 ± 0.23^ab^	0.37 ± 0.02^bc^	244.31 ± 33.18^cdef^
***Avena sativa***	Dumbbell/C_3_	AS	4.4 ± 0.23^ab^	14.1 ± 2.37^d^	0.20 ± 0.02^a^	0.34 ± 0.03^c^	478.95 ± 30.05^ab^
*Nicotiana tabacum*	Elliptical/C_3_	NT	6.9 ± 1.28^abc^	6.5 ± 0.57^abcd^	5.91 ± 1.39^bc^	0.13 ± 0.01^c^	316.21 ± 10.92^bcde^
*Solanum lycopersicum*	Elliptical/C_3_	SL	9.3 ± 1.64^bcd^	4.4 ± 0.90^abc^	3.27 ± 0.22^abc^	0.10 ± 0.01^c^	286.12 ± 22.78^bcde^
*Arabidopsis thaliana*	Elliptical/C_3_	AT	9.9 ± 0.69^bcd^	3.4 ± 0.61^abc^	1.0 ± 0.56^ab^	0.11 ± 0.01^c^	307.91 ± 8.69^bcde^
***Triticum aestivum***	Dumb‐bell/C_3_	TA	11.7 ± 1.13 ^cd^	11.8 ± 2.54 ^cd^	1.03 ± 0.69^a^	0.13 ± 0.02^c^	482.98 ± 18.66^ab^
*Phaseolus vulgaris*	Elliptical/C_3_	PV	12.6 ± 2.08^cde^	11.4 ± 6.10a^bcd^	3.75 ± 1.20^abc^	0.10 ± 0.01^c^	275.42 ± 23.58^cde^
*Pisum sativum*	Elliptical/C_3_	PS	13.2 ± 1.18^cde^	7.9 ± 1.04^abcd^	0.26 ± 0.03^a^	0.04 ± 0.00^c^	209.78 ± 18.34^def^
*Helianthus annuus*	Elliptical/C_3_	HA	14.2 ± 0.67^de^	4.7 ± 0.35^abc^	0.33 ± 0.03^a^	0.09 ± 0.00^c^	446.25 ± 25.52^abc^
*Ginkgo biloba*	Elliptical/C_3_	GB	18.3 ± 2.07^ef^	7.3 ± 0.85^abcd^	6.13 ± 2.53^c^	0.01 ± 0.00^c^	45.20 ± 9.10^f^
*Vicia faba*	Elliptical/C_3_	VF	23.4 ± 2.68^f^	7.9 ± 0.75^abcd^	0.29 ± 0.07^a^	0.08 ± 0.02^c^	430.14 ± 55.49^abcd^

*k*_i_ and *k*_*d*_, time constants for stomatal opening and closing, respectively; λ, initial lag time in response to an increase in irradiance; *Sl*_max_, maximum slope of the temporal response of *g*_*s*_; *G*, steady‐state target reached under 1000 μmol m^−2^ s^−1^ PPFD. The data are means ± SE (*n* = 3–8). Lowercase letters refer to significant differences (*P *<* *0.05) between species (Tukey–Kramer honest significant difference). Species in bold have dumbbell‐shaped guard cells, underlined species have a C_4_ metabolism and species in plain font have elliptical‐shaped guard cells and C_3_ metabolism.

After PPFD was returned to 100 μmol m^−2^ s^−1^, *A* decreased immediately whereas *g*_s_ showed a slow, exponential and species‐dependent decrease. Most species showed a temporal response of *g*_s_ to decreasing light which was an order of magnitude lower than that of *A*, with some exceptions. *Sorghum bicolor* and *Z. mays* demonstrated significantly lower values of *k*_d_ (< 1 min; Fig. 3c) and thus faster responses compared to other species, approaching the speed of assimilation rate response to light (Fig. 2). When PPFD was returned to 100 μmol m^−2^ s^−1^, no significant differences in λ were observed (data not shown), although steady‐state *g*_s_ and *Sl*_max_ varied significantly amongst species.

Significant differences in the opening (*k*_i_) and closing (*k*_d_) time constants were observed amongst species (Fig. 3c); *k*_i_ ranged from 0.9 min in *O. sativa* to 23 min in *V. faba*, and *k*_d_ ranged from 0.9 min in *S. bicolor* to 14 min in *P. vulgaris*. *k*_i_ and *k*_d_ were positively correlated in species with elliptical‐ (*R* ^2^ = 0.29, *P *<* *0.01) and dumbbell‐shaped (*R* ^2^ = 0.52; *P *<* *0.001) guard cells. Although the majority of species showed tendencies for greater rapidity in stomatal closing than opening (Fig. 3c), this was significant in only six species (Fig. 3c). On average, the species with dumbbell‐shaped guard cells were 10 min faster in opening than elliptical species, reaching *G*_smax_ in significantly shorter periods of time (*t*_(44)_ = −8.2, *P *>* *0.0001). C_4_ species increased *g*_s_ more rapidly than C_3_ species (*P *<* *0.0001). Estimations from the closing response showed that dumbbell‐shaped guard cells were also faster than elliptical‐shaped guard cells (*P* < 0.04) and C_4_ species closed faster than C_3_ species (*P *<* *0.0001) (Table 3).

### *g*_s_ limitation of *A*

**Figure 4:**
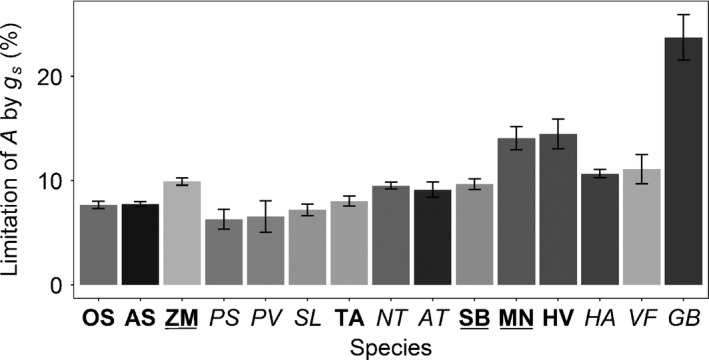
Percentage limitation of net CO_2_ assimilation (*A*) by stomatal conductance (*g*_s_) after the 60 min at 1000 μmol m^−2^ s^−1^ PPFD. Data are the mean ± SE (*n* = 3–5). Species in bold have dumbbell‐shaped guard cells, species underlined have a C_4_ metabolism and species in plain font have elliptical guard cells and C_3_ metabolism (see Table 3 for species name abbreviations).

In order to assess the extent that *A* was limited by *g*_s_ during the increase of PPFD, we determined the time taken to reach 95% of maximum *A* (*A*_95_) at 1000 μmol m^−2^ s^−1^ PPFD (Fig. 3d). Further increases in *g*_s_ after *A*_95_ were substantially greater than the remaining 5% increase in *A,* suggesting that *g*_s_ was no longer limiting *A* (Fig. 2). Stomata opening did not increase significantly after *A*_95_ had been reached for the species with dumbbell‐shaped guard cells (*Z. mays*,* S. bicolor, M. nepalensis, O. sativa*) and for *G. biloba* (Fig. 2). The majority of species achieved *A*_95_ within 30 min with values ranging between 5.7 and 30.7 μmol m^−2^ s^−1^ (Fig. 3b,d). *Zea mays* and *A. sativa* both attained the highest *A* and achieved *A*_95_ in the least time (12–11 min; Fig. 3d), whereas *G. biloba* was the slowest, taking on average 48 min to achieve the lowest *A*_95_. Species with elliptical‐shaped guard cells achieve significantly lower *A*_95_ (*P *<* *0.0001) compared with species with dumbbell‐shaped guard cells (Fig. 3b). The percentage of stomatal limitation of *A* during the opening response to *A*_95_ (Fig. 4) demonstrated significant variation among species (*P *<* *0.0001, *F*_(14,49)_ = 27.368), with values ranging from 6.5 (*P. sativum*) to 24.3% (*G. biloba*). With the exception of *G. biloba,* which was statistically different from all other species, the limitation was 10–15%. Values of *A*_95_ also provided a set point from which to quantify the *g*_s_ response after *A* achieved near maximum steady state (Fig. 2, dotted line) with the majority of species with elliptical‐shaped stomata showing an ‘overshooting’ in stomatal opening, demonstrated by a significant increase in *g*_s_ (*P *<* *0.01) between 10 and 125 mmol m^−2^ s^−1^ (Fig. 3a). Species with dumbbell‐shaped stomata (with the exception of *T. aestivum*;* A. sativa*) and *G. biloba* showed no or only a small ‘overshoot’ (< 3 mmol m^−2^ s^−1^ (Fig. 3a)).

### Quantifying *W*_i_ responses to a step change in PPFD

**Figure 5:**
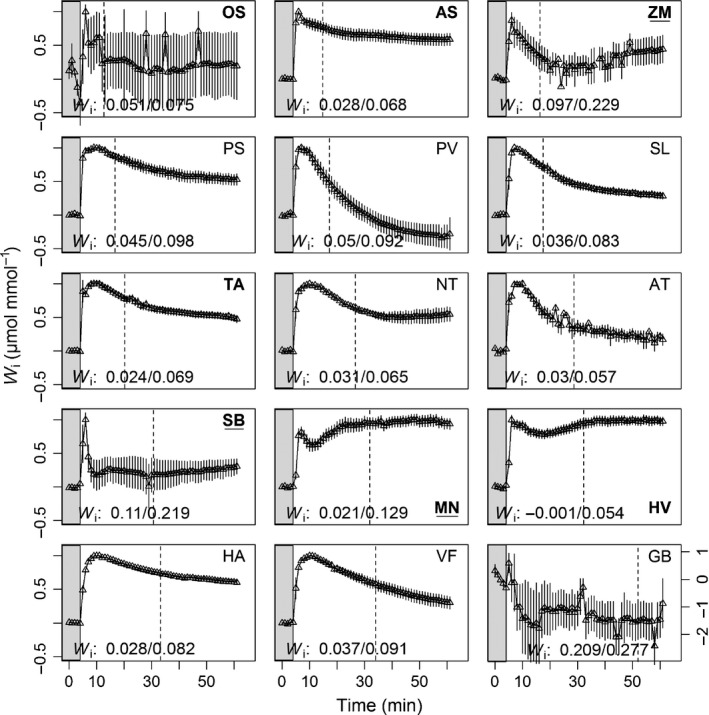
Normalized temporal response of intrinsic water‐use efficiency (*W*_i_) of 15 species to an increase in irradiance from 100 (shade area) to 1000 (white area) μmol m^−2^ s^−1^. Data are the mean ± SE (*n* = 3–5). The initial and maximal average values of *W*_i_ are indicated above the *x*‐axis for each species and a dashed line denotes net CO_2_ assimilation rate at 95% of maximum (*A*_95_). Values were normalized to the initial values at 100 μmol m^−2^ s^−1^ PPFD and maximum values at (1000 μmol m^−2^ s^−1^ PPFD) (see Table 3 for species name abbreviations).

The consequence of the lack of synchrony between the responses of *A* and *g*_s_ to a step increase in PPFD can be illustrated by the temporal responses of *W*_i_ (Figs 5, S4). Following the step increase in PPFD, *A* rapidly increased compared to *g*_s_ (Fig. 2) and *W*_i_ reached a maximum value (*W*_imax_) well before *A*_95_ was achieved. The first 20 min of this response is shown in Fig. S4. The subsequent further increases in *g*_s_ drove a continuous decrease in *W*_i_ until both *A* and *g*_s_ reached steady state. In most of the C_3_ species, *W*_i_ continued to decrease after *A* had reached a steady state due to the continued increase in *g*_s_. *W*_imax_ represents the greatest CO_2_ uptake for *g*_s_; however, it should be noted that this value occurs earlier in the transient response before *A*_95_ is reached and that *W*_imax_ is achieved only for an extremely brief period of time. The diversity in *W*_i_ between species was determined by *G*_smax_ rather than *A*_max_ as no correlation between *A*_max_ with *W*_imax_ was observed, whereas *G*_smax_ was negatively correlated with *W*_imax_ (*P *<* *0.0001, *r*_s_ = −0.67) and with *W*_i_ before the decrease in PPFD (*P *<* *0.0001, *r*_s_ = −0.74). For example, *G. biloba*,* S. bicolour, M. nepalensis* and *Z. mays* achieved and maintained the highest *W*_i_ (*P *<* *0.001) with vastly different values of *A* by maintaining a relatively low *g*_s_ compare to other species. The balance between CO_2_ fixation and water loss was different between species, revealed by the four‐fold difference in *W*_imax_ observed between the 15 species, with the lowest values observed in *H. vulgare* (0.054 ± 0.006 μmol mmol^−1^ m^−2^ s^−1^). On average, the percentage decrease between *W*_imax_ and *W*_i_ at the end of the response under 1000 μmol m^−2^ s^−1^ PPFD was significantly less in species with dumbbell‐shaped guard cells (*P *<* *0.0001) than in species with elliptical‐shaped guard cells.

**Figure 6:**
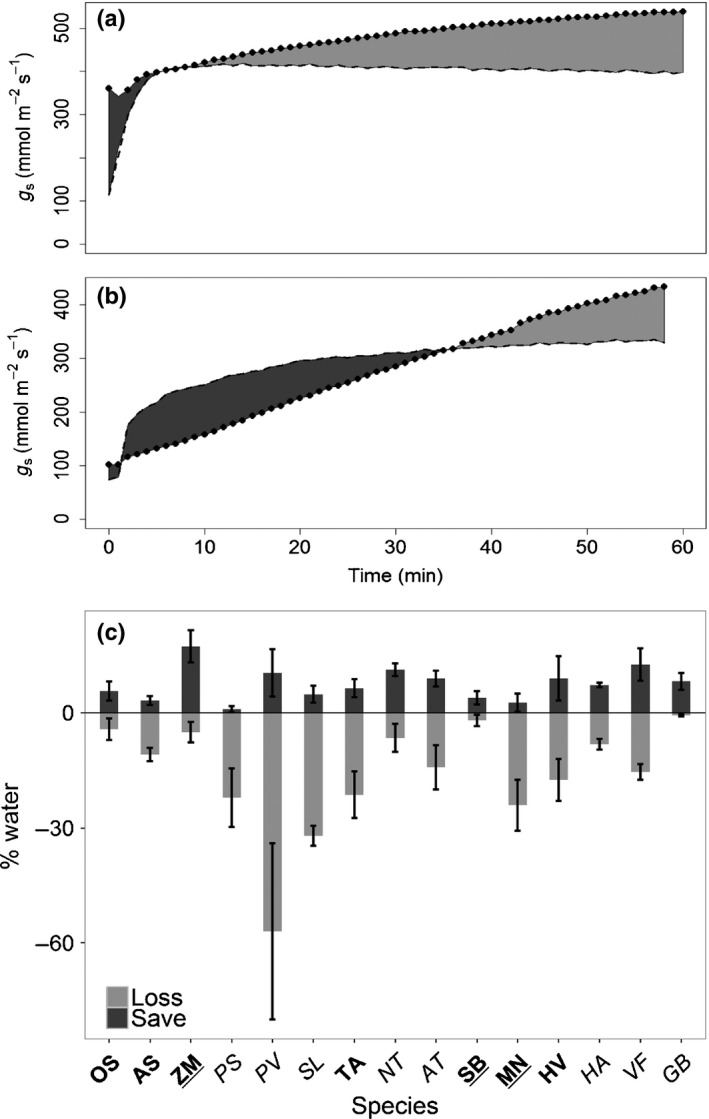
Examples of observed (dotted lines) and modelled (dashed lines) temporal response of stomatal conductance to water vapour (*g*_s_) for (a) wheat (*Triticum aestivum*) and (b) broad bean (*Vicia faba*). The modelled data represent *g*_s_ at constant water‐use efficiency (*W*_i_) achieved at 95% net CO_2_ assimilation (*A*_95_). The light grey shading represents water loss and the dark grey shading represents water conserved. (c) The percentage change in water loss (light grey) and water conserved (dark grey) for a 5% increase in *A* derived from the differences in observed and modelled for each species. Data are the mean ± SE (*n* = 3–5). Species in bold have dumbbell‐shaped guard cells, underlined species have a C_4_ metabolism and species in plain font have elliptical‐shaped guard cells and C_3_ metabolism (see Table 3 for species name abbreviations).

The temporal response of *W*_i_ is driven by the temporal variation in *g*_s_ to increasing PPFD that is uncoordinated with *A*, resulting in unnecessary water loss (Fig. 2). To investigate the theoretical variation of *g*_s_ required to optimize *W*_i_, if coordinated with *A*, a model with a constant *W*_i_ chosen at *A*_95_ (*W*_i95_) was applied and the difference between observed and modelled *g*_s_ assessed (Fig. 6), with modelled values of *g*_s_ greater than observed signifying ‘water saving’ and values less than observed representing a ‘water loss’. Figure 6(a,b) show examples for *T. aestivum* and *V.faba*. During the first part of the response, the amount of potential ‘water saved’ was not significantly different between species (0.98–17.3%) (Fig. 6c). However, after *W*_i95_ was achieved, a significant difference in ‘water loss’ between species, in terms of percentage change in *g*_s_ (*P *<* *0.0001, *F*_(14,49)_ = 3.454) was observed. For example, in *P. vulgaris, g*_s_ increased by 57% (± 23%) for only a 5% gain in *A*, which illustrates the strong uncoupling of *A* and *g*_s_ in this species and the negative impact on *W*_i_ (Figs 3a, S5). By contrast, the observed response of *g*_s_ in *S. bicolor* was close to the modelled optimal *g*_s_ with minimal increases in *g*_s_ once *A*_95_ was reached (Figs 3a, 6c).

The results revealed that *W*_imax_ and *A*_95_ were not reached at the same point during the temporal response (Fig. 5). To reach *A*_95_ (denoted by the dotted line, Fig. 5), the species typically displayed a decrease in *W*_i_ from *W*_imax_. The percentage increase in *A* from 100 to 1000 μmol m^−2^ s^−1^ PPFD was significantly greater (*P *<* *0.01) than the percentage decrease in *W*_i_. The highest gains in *A* were observed for *G. biloba* and the species with dumbbell‐shaped guard cells (with the exception of *M. nepalensis*) which all achieved > 30% increase in *A* (Fig. S5).

### Anatomical features

Stomatal density was significantly different between species with abaxial stomatal densities ranging from 68.5 to 376.3 mm^−2^ and adaxial densities between 0 and 281.6 mm^−2^ (Fig. S6) A positive correlation between ad‐ and abaxial density for species both with elliptical‐ (*R* ^2^ = 0.87) and dumbbell‐shaped (*R* ^2^ = 0.79 excluding *M. nepalenis*) guard cells was observed. When considering both types of guard cells, a strong correlation between abaxial and adaxial values for stomatal density (*R* ^2^ = 0.76 excluding *M. nepalensis*), pore length (PL; *R* ^2^ = 0.79) and guard cell width (GCW; *R* ^2^ = 0.85) was also observed, and therefore mean values were used to correlate with stomatal response traits. A strong correlation between PL and GCW was observed and hence only PL was used for further analyses.

With reference to opening responses of elliptical‐shaped guard cells, no significant relationships were found between the anatomical features (PL and SD) and *Sl*_max_, *k* or G, whereas in dumbbell‐shaped guard cells, PL and SD correlated significantly with *G*_smax_, and *k*_i_ was correlated with PL but not with SD. The same correlations were observed with reference to closing responses in both guard cell types; however, a significant relationship between SD and *k*_i_ was also observed in dumbbell‐shaped guard cells (Table 2).

## Discussion

As light changes rapidly and is often considered the most dynamic and most important environmental variable influencing both stomatal behaviour and photosynthetic rate, we examined the kinetics of photosynthesis (*A*) and stomatal conductance (*g*_s_) to a step increase followed by a decrease in photosynthetic photon flux density (PPFD), in a number of species; assessing the speeds of the *g*_s_ response, the amplitude of change, *g*_s_ limitation of *A* and the impact of these kinetics on intrinsic water use efficiency (*W*_i_). The temporal dynamics showed clear species‐specific differences and noncoordination between *A* and *g*_s_, with *g*_s_ exhibiting a slower and more varied response than *A*. Such uncoordinated *A* and *g*_s_ responses could have significant implications for cumulative carbon assimilation and transpirational water loss, especially in dynamic light environments. For example, Lawson & Blatt (2014) modelled synchronous *g*_s_ and *A* behaviour and calculated a theoretical 20% increase in water use efficiency if *g*_s_ responded instantaneously to the changes in PPFD and matched mesophyll demands for CO_2_.

A combination of rapid responses and high steady‐state values of *g*_s_ reduce CO_2_ diffusional limitations of *A*, but can also drastically reduce *W*_i_, due to the nonlinear relationship between *A* and *g*_s_ (Wong *et al*., 1979). We show for example that the high steady‐state values and rapid responses observed in *Oryza sativa*,* Avena sativa* and *Triticum aestivum* facilitated high photosynthetic rates but ultimately resulted in low *W*_i_, which may be indicative of traditional breeding and selection practices for high yield at the expense of water loss (Jones, 1987). Although high *g*_s_ reduces *W*_i_, it is also possible that, under well‐watered conditions, such stomatal behaviour would increase overall photosynthetic carbon gain by enabling plants to opportunistically use sun flecks in the canopy that can occur on a timescale of seconds to hours (Chazdon & Pearcy, 1991; Kirschbaum *et al*., 1988; Pearcy, 1990; Way & Pearcy, 2012). Under the measurement conditions used here, when PPFD was raised *A* immediately (within 1 min) increased in all species, indicating that *g*_s_ at the lower light level was greater than required. However, a clear stomatal limitation of *A* was also apparent as all species took > 9 min to reach 95% final *A* (*A*_95_) (Fig. 3d).

A common feature of *g*_s_ dynamics was the noncoordination in *A* and *g*_s_ responses and the continued stomatal opening after *A*_95_ had been reached (or ‘overshooting’ of *g*_s_; Fig. 3a), resulting in decreases in *W*_i_. The observed diversity in responses of *A* and *g*_s_ in the species measured questions the mechanisms that coordinate these parameters. Intercellular CO_2_ concentration (*C*_i_) was originally proposed as the mediator for the close correlation between *g*_s_ and *A* (Wong *et al*., 1979; Farquhar & Wong, 1984; Mansfield *et al*., 1990; Buckley *et al*., 2003). It was assumed that stomata adjust to a steady‐state aperture to maintain *C*_i_ at 2/3 atmospheric [CO_2_] (Ehleringer & Pearcy, 1983) and therefore, when *A* is increasing the resulting decrease in *C*_i_ would cause stomata to open and *vice versa*. When *A* reaches steady state, further increases in *g*_s_ would result in a greater *C*_i_ that cannot increase *A* and therefore, following the *C*_i_ hypothesis, no further increases in *g*_s_ would be expected once steady‐state *A* has been achieved. However, our results do not fully support this conclusion (e.g. *Vicia faba* in Fig. 2) and agree with findings from work on transgenic plants with reductions in photosynthesis, which showed increasing *g*_s_ with light despite high *C*_i_ (Von Caemmerer *et al*., 2004; Baroli *et al*., 2008; Lawson *et al*., 2008). Many studies support *C*_i_‐driven stomatal responses (e.g. Roelfsema & Prins, 1995) and we do not argue against CO_2_ as a driver; however, our results show that *C*_i_ is clearly not of high priority in the hierarchy.

Stomata have been a key target for improving plant water use efficiency (WUE) and/or a plant's ability to cope with reductions in water availability. However, improvements of WUE in crop plants often come at the expense of photosynthetic rates (Yoo *et al*., 2009, 2010) and are therefore of limited value, given that current global research efforts focus on increasing crop yield for sustainable food and fuel production (Long *et al*., 2015). However, as we have illustrated here, *W*_imax_ does not correspond to maximum assimilation rate (Fig. 5) as maximum WUE can often only be achieved when *g*_s_ restricts *A*. Based on these observations of dynamic responses we could suggest a steady‐state *g*_s_ target that would provide a compromise between *A* and *W*_i_ and propose that this target should be the lowest *g*_s_ value that enables *A*_95_ to be achieved. It should be noted that this is an optimal target and that fluctuations in the environment could result in different integrated values of *A*,* g*_s_ and therefore *W*_i_, highlighting the importance of appreciating the speed of stomatal responses and coordination between *A* and *g*_s_.

It is well known that significant variation in photosynthetic capacity (*A*_max_) exists both within and amongst different species (Lawson *et al*., 2012) and, as observed here, this is generally correlated with steady‐state *g*_s_ (and *G*_smax_). As may be expected the C_4_ species measured in our study were able to achieve a greater *A*_95_ at a lower *g*_s_ (at *A*_95_) compared to C_3_ species (Fig. S3), and it is likely that the faster stomatal opening and closing responses observed in C_4_ species (Fig. 3c) facilitated this greater level of coordination between *A* and *g*_s_ (Fig. 2). This faster response was a common feature not just in C_4_ plants, but also C_3_ species with dumbbell‐shaped guard cells. However, despite the close coupling in C_4_ species, the same stomatal limitation on *A* of *c*. 10% or greater was observed in both C_4_ and C_3_ plants (Fig. 4). This illustrates the importance of considering both CO_2_ uptake and water loss when evaluating steady‐state or transient W_i_ (McAusland *et al*., 2013), as maximum *W*_i_ is often not observed at maximum *A*. Here the decrease in *W*_i_ (between W_imax_ and *A*_95_) with increasing *g*_s_ was outweighed by a substantial gain in *A* in all species.

The temporal uncoupling between stomatal behaviour and carbon demand observed in many species can be evaluated by comparing measured *g*_s_ responses with those modelled assuming a stable *W*_i_ (at *A*_95_, which represented a *W*_i_ value that is achieved without a limitation on *A*), providing an estimate of the differences between variable and stable *W*_i_ in terms of water gain and expense for each species. Using this model over the period measured, stomatal behaviour in the majority of species resulted in water expense exceeding water conservation, illustrating that the latter was not the priority. As all the plants in these experiments were maintained under relatively well‐watered conditions, this would have led to a higher stomatal conductance than would be observed in plants experiencing water limitation (Comstock & Ehleringer, 1993; Mott & Peak, 2012; Lawson & Blatt, 2014). In general, C_4_ species were an exception to this and either demonstrated a balanced water budget or greater gains than losses, further exemplifying the more synchronous *A* and *g*_s_ responses observed in the three species measured. The most likely explanation for the greater loss of water in C_3_ species is the substantial overshoot in *g*_s_ after *A*_*95*_ had been achieved, which was not apparent in the two C_4_ species studied (Figs 3a, 6).

However, C_3_ species with rapid stomata responses (e.g. *O. sativa*) also exhibited a positive water balance, whilst species with the slowest stomatal opening (with the exception of *G. biloba*) demonstrated the most negative water balances (Fig. 6c) hinting at the possible existence interspecific diversity of stomatal control.

The rapidity of response for stomata to open (increase; *k*_i_) and close (decrease; *k*_d_) was positively correlated for species with elliptical‐ as well as dumbbell‐shaped guard cells, suggesting that similar mechanisms or pathways were involved in both opening and closing responses. Overall, significant asymmetry of the stomatal responses revealed a faster closing than opening, which has previously been associated with conserving water (Tinoco‐Ojanguren & Pearcy, 1993; Ooba & Takahashi, 2003). In comparison, species with dumbbell‐shaped guard cells displayed the fastest responses and most had greater similarity in the rapidity of opening and closing, which is consistent with the fact that these guard cells require fewer solutes and less water to achieve a given unit increase in aperture (Franks & Farquhar, 2007; Raven, 2014). The rapidity of increasing *g*_s_ impacts on *A,* with species with high *k*_i_ taking longer to achieve *A*_95_, as low *g*_s_ restricts CO_2_ diffusion. Under field conditions with a dynamic light environment, slowly responding stomata could restricted CO_2_ uptake and thus have a compound effect on the cumulative *A* over the growing season and affect yield (Reynolds *et al*., 1994; Fischer *et al*., 1998). However, slow stomatal closure would negatively impact on *W*_i_ when environmental conditions reduce *A*. It should be noted that under field conditions, changing the light environment also results in changes in leaf temperature. A direct impact of increasing PPFD would be an increased leaf temperature, which would lead to higher leaf‐to‐air vapour pressure difference and thus exacerbate the transpirational losses of ‘overshooting’ stomata. However, higher transpirational losses would have a cooling effect. Therefore, concomitant temperature variation could have complex effects on the dynamic responses of *A*,* g*_s_ and *W*_i_, and should be studied in detail using appropriate experimental set‐ups.

Variation between species was also observed in the maximum speed of *g*_s_ response (*Sl*_max_) and the rapidity of opening (*k*_i_) to achieve steady‐state conductance. Previous research has associated the speed of stomatal responses with the size of stomata, with smaller stomata facilitating rapid opening and closing (Hetherington & Woodward, 2003; Franks & Farquhar, 2007; Franks & Beerling, 2009; Drake *et al*., 2013; Raven, 2014).The majority of these studies have used the maximum slope (*Sl*_max_) as a measure of the maximum speed of response; however, this measurement is also dependent on the amplitude of the response (Eqn (Eqn 2)). Additionally, because *g*_s_ is determined by both stomatal aperture and density, small changes in aperture in plants with smaller, more numerous stomata will have a greater *Sl*_max_ for the same change in aperture as species with fewer larger stomata. Therefore, although *Sl*_max_ may provide a useful comparative measure within species (in which anatomical features and the scales of stomatal responses are similar), it is not a useful parameter to compare speeds of response between species with different anatomical features and magnitudes of change. To address this issue, we used the time constant *k* to provide a measure of the rapidity of *g*_s*,*_ independently of the magnitude of response and the absolute *g*_s_ values observed. For elliptical‐shaped guard cells, we did not detect any significant correlation between stomatal density and *Sl*_max_ or *k*, suggesting that on an interspecific basis neither the speed nor the amplitude of the stomatal responses to PPFD were dependent on stomatal density. On the other hand, for dumbbell‐shaped guard cells, variation among species in stomatal size impacted on the speed and amplitude of stomatal responses. This hints at the possibility that for elliptical‐shaped guard cells attributes other than anatomical features are important contributors to the speed of stomatal responses (Hetherington & Woodward, 2003; Franks & Beerling, 2009) such as membrane permeability due to ion channels number or distribution (see discussion, Lawson & Blatt, 2014). By contrast, for dumbbell‐shaped guard cells, anatomical variations seem to impact the rapidity of their response. Additionally, these species were also able to achieve a greater *Sl*_max_ and tended to be faster. This may be due to the energetic requirements for stomatal movement of the often smaller dumbbell‐shaped guard cells (Grantz & Assmann, 1991; Franks & Farquhar, 2007; Raven, 2014). The dumbbell‐shaped design means that small changes in width can cause larger changes in stomatal aperture and maximize the potential of these stomata to track changes in environmental conditions (Hetherington & Woodward, 2003).

Although transients of leaf‐level *W*_i_ provide insight into potentially optimizing stomatal behaviour, numerous other processes contribute to *W*_i_ in the field. Manipulation of the speed of *g*_s_ provides scope for improving carbon acquisition in fluctuating light environments but also enhances drought tolerance through improved conservation of water. Integration of these dynamic responses over daily or seasonal time periods is complex and would require a model that includes respiration (both from leaves and parts including stems and roots) and transpiration as a product of changes in diurnal saturation deficit (Cowan & Farquhar, 1977; Farquhar *et al*., 1989; Jones, 2004) Identifying varieties or genotypes with more rapid stomatal responses could be used as an optimizing strategy for whole‐plant water use over the growing period, potentially improving the ability of the plant to adapt to changing environments (Schulze & Hall, 1982; Campitelli *et al*., 2016) which could feed forward to maintain or improve yields (Chaerle *et al*., 2005; Lawson & Blatt, 2014).

### Conclusion

This is one of the few studies to investigate temporal responses in *A* and *g*_*s*_ in relation to carbon assimilation and *W*_i_, and illustrates significant species‐specific variation in the speed of stomatal responses and magnitude of change, as well as coordination with *A*. Slow stomatal responses can limit *A* by *c*. 10%, which could equate to substantial losses in photosynthetic rates, productivity and reductions in yield. Previous research focusing on improving productivity has shown that by enhancing photosynthesis by only 2–3%, substantial increases in plant growth and biomass can be achieved over the season (Lefebvre *et al*., 2005; Zhu *et al*., 2007; Simkin *et al*., 2015). The work presented here illustrates that similar short‐term improvements in *A* could be gained by improving the rapidity of stomatal responses and coordination with *A*. Tighter coupling between stomata and *A* therefore has the potential to achieve a substantial improvement in WUE, as in the present study, overshooting of *g*_*s*_ by up to 80% was observed for only a 5% gain in *A* and fast closing responses resulted in substantial saving in water loss.

Our findings support faster responses in dumbbell‐ compared with elliptical‐shaped guard cells and suggest that photosynthetic type (C_3_/C_4_) also plays a role. The speed of stomatal responses might not be dependent on the same underlying processes when comparing elliptic‐ and dumbbell‐shaped guard cells, with physiological processes being more important for the former and anatomical features for the latter. Improving the rapidity of stomatal responses could greatly improve productivity and *W*_i_ but achieving this will require greater knowledge of the physiological and molecular mechanisms that determine the speed of stomata and coordination with mesophyll demands for CO_2_, and further field‐based measurements that integrate the dynamics of *A*,* g*_*s*_ and *W*_i_ over seasons.

## Author contributions

L.M., N.R.B. and T.L. planned and designed the research. L.M., S.V‐C., P.D. and T.L. performed experiments and analysed data, L.M., S.V‐C., P.D., N.R.B., O.B. and T.L. wrote the manuscript.

## Supporting information

Please note: Wiley Blackwell are not responsible for the content or functionality of any supporting information supplied by the authors. Any queries (other than missing material) should be directed to the *New Phytologist* Central Office.

**Fig. S1 **Response of stomatal conductance to water vapour (*g*_s_) of 15 species to an increase in irradiance from 100 to 1000 μmol m^−2^ s^−1^ PPFD.
**Fig. S2 **Response of net CO_2_ assimilation (*A*) of 15 species to an increase in irradiance from 100 to 1000 μmol m^−2^ s^−1^ PPFD.
**Fig. S3 **The relationship between 95% maximum net CO_2_ assimilation (*A*_95_) and steady‐state stomatal conductance under 1000 μmol m^−2^ s^−1^ PPFD (*G*_*s*max_) for 15 species.
**Fig. S4** Normalized temporal response of intrinsic water‐use efficiency (*W*_i_) of 15 species for the first 20 min after an increase in irradiance from 100 to 1000 μmol m^−2^ s^−1^.
**Fig. S5** Determining the percentage decrease in intrinsic water‐use efficiency (*W*_i_) for a percentage increase in CO_2_ assimilation (*A*) between maximum *W*_i_ max to 95% of the maximum *A* (*A*_95_) reached under 1000 μmol m^−2^ s^−1^ PPFD for 15 species.
**Fig. S6** Counts of stomatal density and measurements of guard cell length and width for 15 species from the adaxial and abaxial surfaces of the leaf.
