# Supplementary material for: Effects of kinetics of light‐induced stomatal responses on photosynthesis and water‐use efficiency
Source: New Phytol. 2016 May 23;211(4):1209–20. doi: 10.1111/nph.14000 (PMC4982059; doi:10.1111/nph.14000)
Supplement: Supplementary file 1 — Fig. S1 Response of stomatal conductance to water vapour (g s) of 15 species to an increase in irradiance from 100 to 1000 μmol m−2 s−1 PPFD. Fig. S2 Response of net CO2 assimilation (A) of 15 species to an increase in irradiance from 100 to 1000 μmol m−2 s−1 PPFD. Fig. S3 The relationship between 95% maximum net CO2 assimilation (A 95) and steady‐state stomatal conductance under 1000 μmol m−2 s−1 PPFD (G smax) for 15 species. Fig. S4 Normalized temporal response of intrinsic water‐use efficiency (W i) of 15 species for the first 20 min after an increase in irradiance from 100 to 1000 μmol m−2 s−1. Fig. S5 Determining the percentage decrease in intrinsic water‐use efficiency (W i) for a percentage increase in CO2 assimilation (A) between maximum W i max to 95% of the maximum A (A 95) reached under 1000 μmol m−2 s−1 PPFD for 15 species. Fig. S6 Counts of stomatal density and measurements of guard cell length and width for 15 species from the adaxial and abaxial surfaces of the leaf. [file NPH-211-1209-s001.pdf]

New Phytologist

New Phytologist Supporting Information

Article Title: **Effects of kinetics of light-induced stomatal responses on photosynthesis and water-use efficiency**

Authors: Lorna McAusland, Silvère Vialet-Chabrand, Philip Davey, Neil R. Baker, Oliver Brendel and Tracy Lawson

Article acceptance date: 24 March 2016

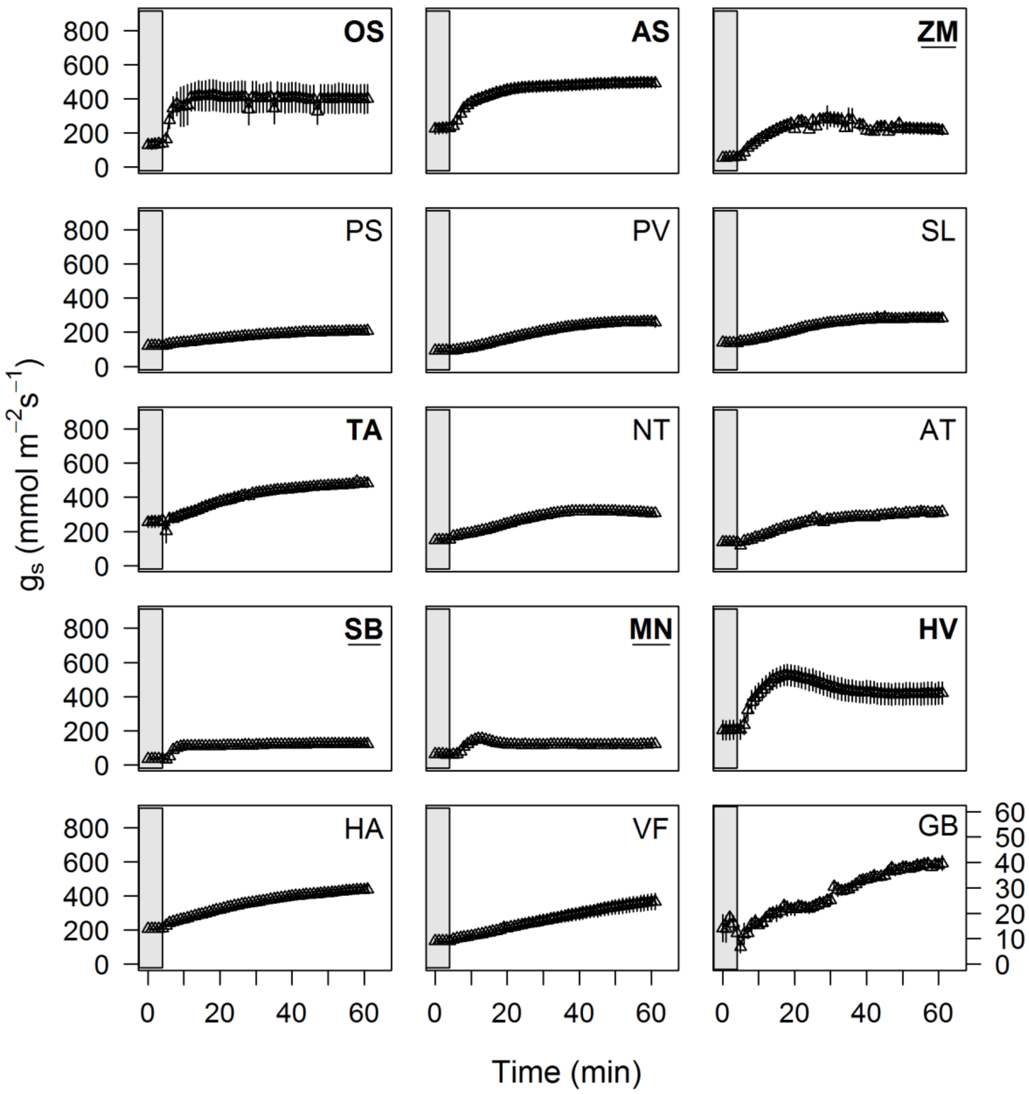

Fig. S1 Response of stomatal conductance to water vapour (g_s) of 15 species to an increase in irradiance from 100 to 1000 µmol m^{-2} s^{-1} PPFD (see Table 3 for abbreviations of species nomenclature). Data are the mean ± SE (n = 3-8).

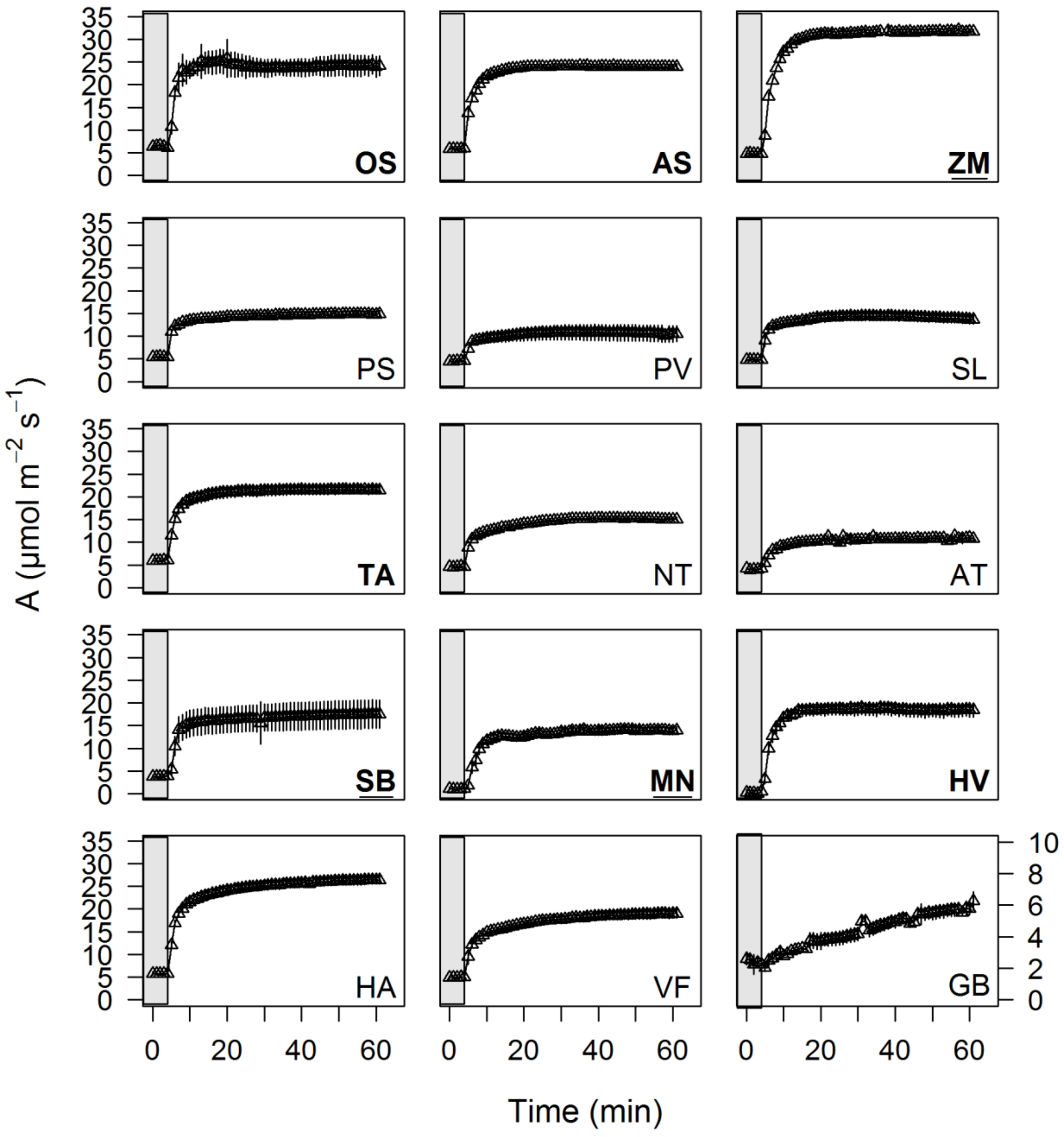

Fig. S2 Response of net CO_2 assimilation (*A*) of 15 species to an increase in irradiance from 100 to 1000 µmol m^{-2} s^{-1} PPFD (see Table 3 for abbreviations of species nomenclature). Data are the mean ± SE (*n* = 3-8).

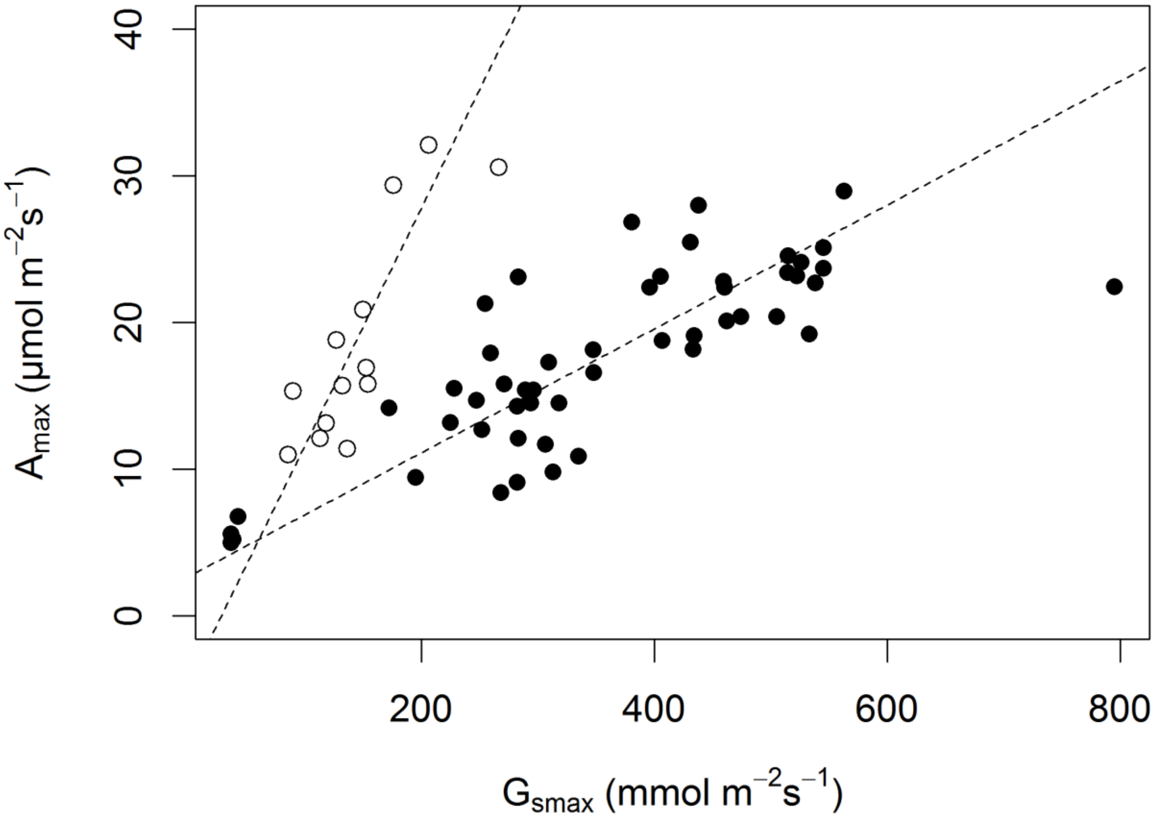

Fig. S3 The relationship between 95% maximum net CO_2 assimilation (*A*95) and steady–state stomatal conductance under 1000 μmol m^{-2} s^{-1} PPFD (G_{smax}) for 15 species, demonstrating C3 (black circle) and C4 (open circle) photosynthetic pathways. Data are individual measurements.

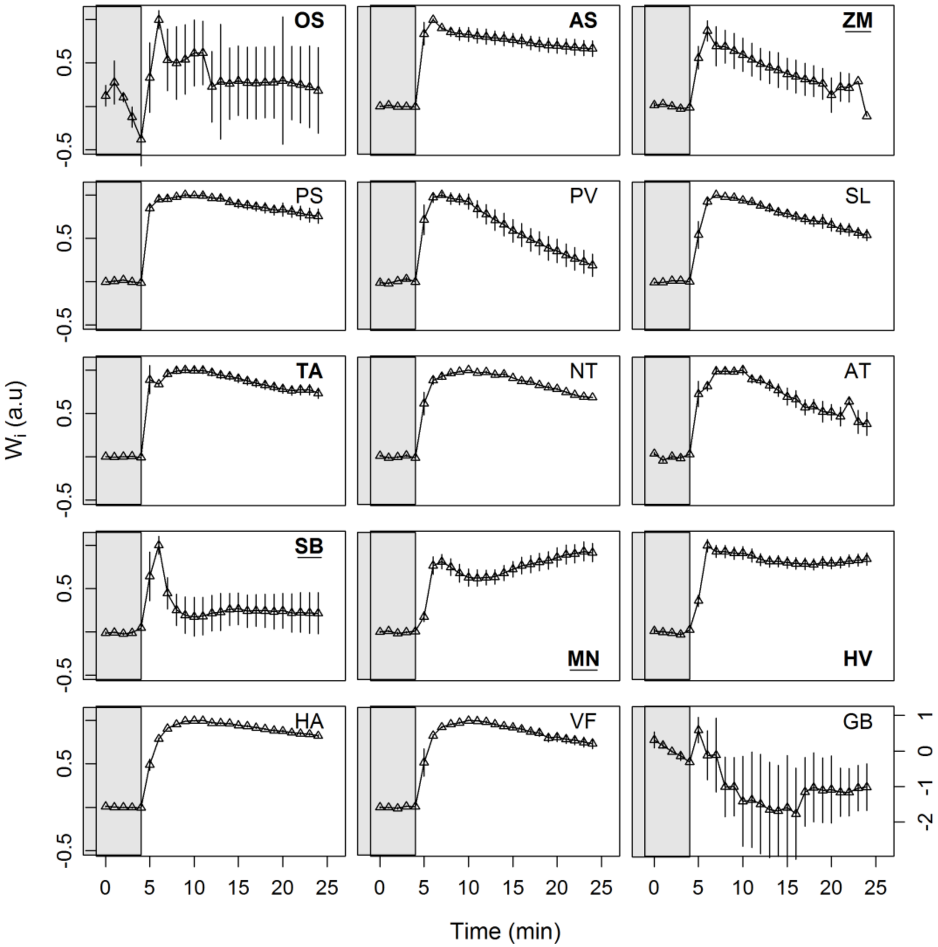

Fig. S4 Normalized temporal response of intrinsic water-use efficiency (W_i) of 15 species for the first 20 min after an increase in irradiance from 100 (shade area) to 1000 (white area) µmol m^{-2} s^{-1}. Data are the mean ± SE (n = 3-5). Values were normalized to the initial values at 100 µmol m^{-2} s^{-1} PPFD and maximum values at (1000 µmol m^{-2} s^{-1} PPFD) (see Table 3 for species name abbreviations).

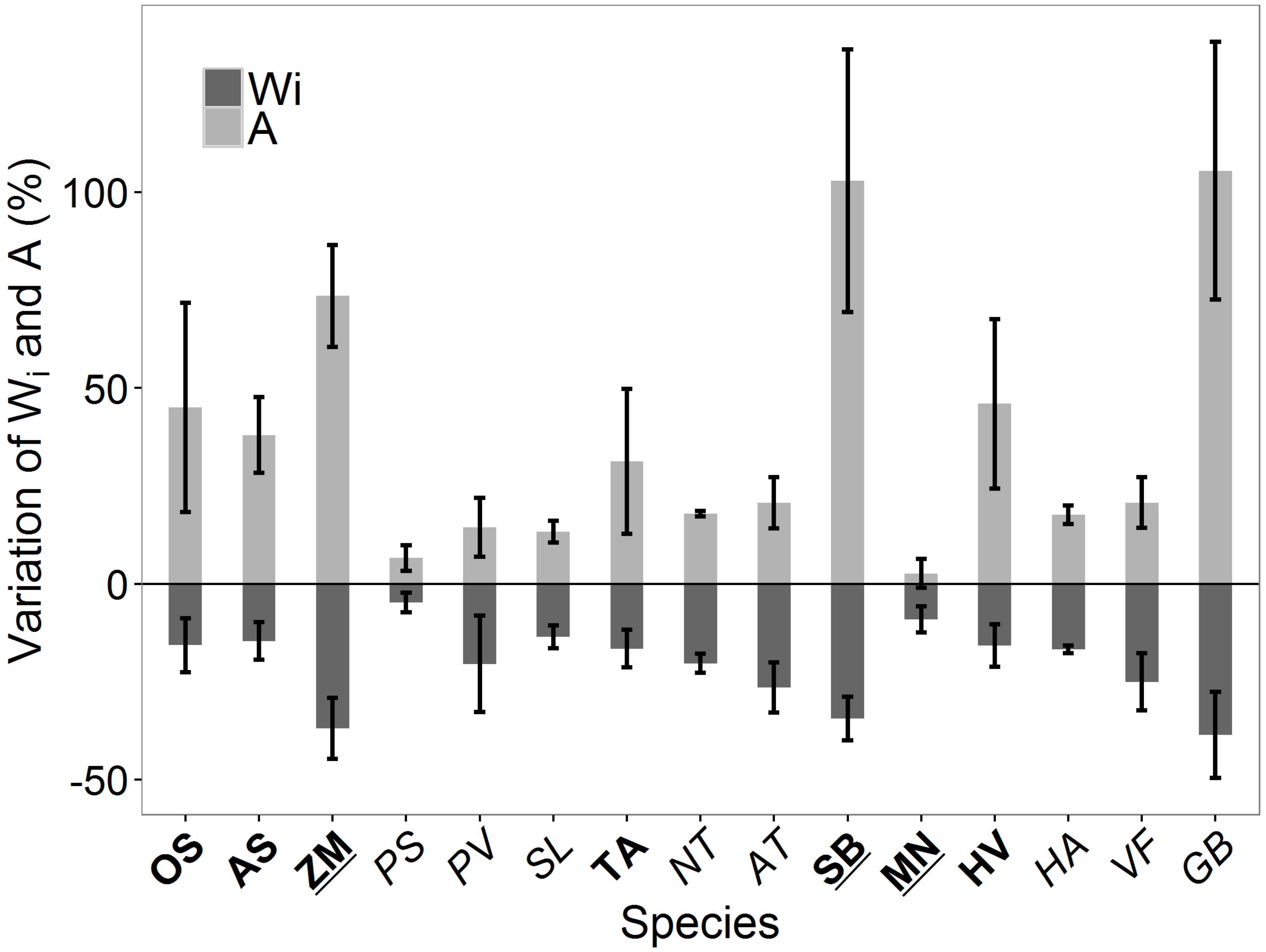

Fig. S5 Determining the percentage decrease in intrinsic water-use efficiency (W_i - dark grey) for a percentage increase in CO_2 assimilation (*A* - light grey) between maximum W_i max to 95% of the maximum *A* (A_{95}) reached under 1000 μmol m^{-2} s^{-1} PPFD for 15 species. Data are the mean ± SE (*n* = 3-8). Species in bold had dumb-bell shape guard cells and species underlined had a C4 metabolism.

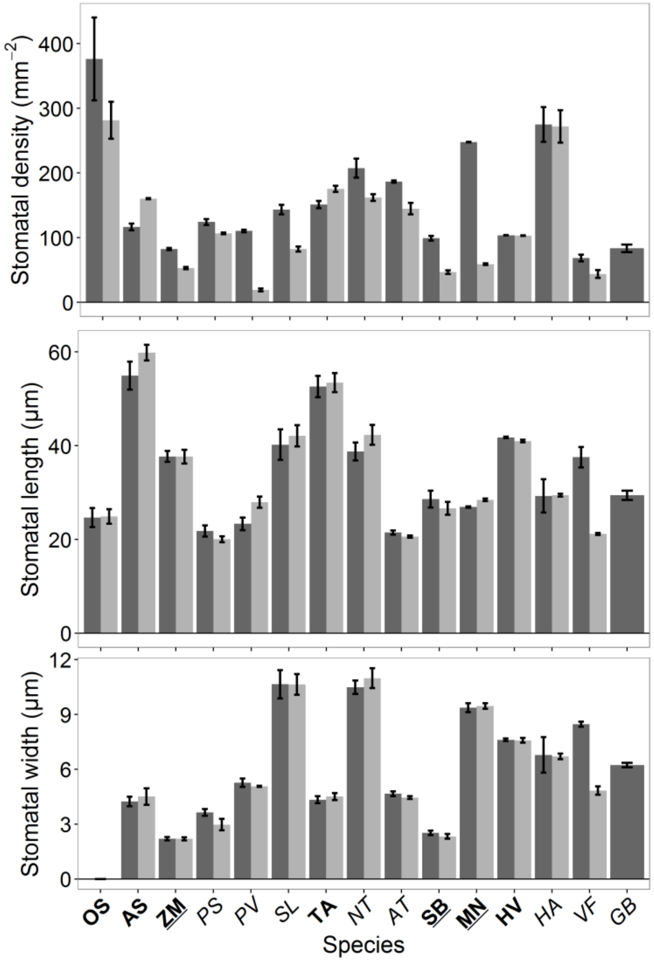

Fig. S6 Counts of (a) stomatal density and measurements of (b) guard cell length and (c) width for 15 species from the adaxial (light grey) and abaxial (dark grey) surfaces of the leaf. Data are the mean ± SE (*n* = 3-8). Species in bold had dumb-bell shape guard cells and species underlined had a C4 metabolism.
